# CuAAC Click Chemistry Accelerates the Discovery of Novel Chemical Scaffolds as Promising Protein Tyrosine Phosphatases Inhibitors

**DOI:** 10.2174/092986712800269245

**Published:** 2012-05

**Authors:** X.-P. He, J. Xie, Y. Tang, J. Li, G.-R. Chen

**Affiliations:** 1 Key Laboratory for Advanced Materials & Institute of Fine Chemicals, East China University of Science and Technology, Shanghai 200237, PR China; 2 National Center for Drug Screening, State Key Laboratory of Drug Research, Shanghai Institute of Materia Medica, Shanghai Institutes of Biological Sciences, Chinese Academy of Sciences, Shanghai 201203, PR China; 3 PPSM, Institut d’Alembert, ENS de Cachan, CNRS UMR 8531, F-94235 Cachan, France

**Keywords:** Protein tyrosine phosphatase, click chemistry, *in situ* screening, drug discovery, CuAAC, tyrosine phosphorylation, dephosphorylation, carbohydrate, amino acid, salicylic acid, isoxazole acid, ketocarboxylic acid, competitive inhibitor, bidentate

## Abstract

Protein tyrosine phosphatases (PTPs) are crucial regulators for numerous biological processes in nature. The dysfunction and overexpression of many PTP members have been demonstrated to cause fatal human diseases such as cancers, diabetes, obesity, neurodegenerative diseases and autoimmune disorders. In the past decade, considerable efforts have been devoted to the production of PTPs inhibitors by both academia and the pharmaceutical industry. However, there are only limited drug candidates in clinical trials and no commercial drugs have been approved, implying that further efficient discovery of novel chemical entities competent for inhibition of the specific PTP target in vivo remains yet a challenge. In light of the click-chemistry paradigm which advocates the utilization of concise and selective carbon-heteroatom ligation reactions for the modular construction of useful compound libraries, the Cu(I)-catalyzed azide-alkyne 1,3-dipolar cycloaddition reaction (CuAAC) has fueled enormous energy into the modern drug discovery. Recently, this ingenious chemical ligation tool has also revealed efficacious and expeditious in establishing large combinatorial libraries for the acquisition of novel PTPs inhibitors with promising pharmacological profiles. We thus offer here a comprehensive review highlighting the development of PTPs inhibitors accelerated by the CuAAC click chemistry.

## 1 INTRODUCTION

Tyrosine phosphorylation (TP) is a fundamental mechanism modulating a number of important physiological processes of eukaryotes such as the communication between and within cells, the change in shape and motility of cells, cell proliferation and differentiation, gene transcription, mRNA processing, and the intra- and intercellular transportations of molecules. TP also plays crucial roles in embryogenesis, organ development, tissue homeostasis, and immune response. As a consequence, abnormalities of TP may cause the pathogenesis of numerous inherited or acquired human diseases.

Reversible tyrosine phosphorylation is governed by the balanced action of protein tyrosine kinases (PTKs) and protein-tyrosine phosphatases (PTPs). Perturbation of PTK activity by mutations or overexpressions results in malignant transformation [1], and PTK inhibitors are established as anticancer drugs [2]. However, it has recently become apparent that protein phosphatases can no longer be viewed as passive housekeeping enzymes in these processes. In fact, the PTPs constitute a large family of enzymes that parallel tyrosine kinases in their structural diversity and complexity. There are 107 PTP members decoded from the human genome and they can be classified further into four families: classes I, II and III of cysteine-based PTPs and the aspartate-based PTPs. Within the class I PTPs, there are 38 phosphotyrosine-specific enzymes referred to as the ‘classical PTPs’ and 61 dual-specific phosphatases that dephosphorylate both serine/threonine and tyrosine residues [3]. Compared to the 90 human PTK genes, a similar level of complexity between the two families is suggested. However, the number of genes only illustrates the minimal level of complexity as additional diversities are also introduced through the use of alternative promoters, alternative mRNA splicing and post-translational modifications. This is indicative of the functional importance of PTPs in the control of cell signaling. Recently, biochemical and genetic studies indicate that protein phosphatases can exert both positive and negative effects on signaling pathways, and play crucial physiological roles in a variety of mammalian tissues and cells [4, 5].

## 2 PTPS AS DRUG TARGETS

Malfunction of PTPs has been demonstrated to link with the pathogenesis of various human diseases including cancers, diabetes, obesity, autoimmune disorders, and neurodegenerative diseases [6, 7]. Consequently, the PTPs offer a wealthy class of drug targets for the development of novel chemotherapeutics. Among this large superfamily, protein tyrosine phosphatase 1B (PTP1B) represents the best validated drug target. This enzyme can dephosphorylate activated insulin receptor (IR) or insulin receptor substrates (IRS), and JAK2 that is the downstream of leptin receptor. Subsequent research indicated that PTP1B knockout mice display improved insulin sensitivity and glycemic control, and are resistant against weight gain with much lowered triglyceride level [8, 9]. Moreover, recent biochemical studies established that PTP1B also functions as an oncogene in the context of breast cancer [10]. As a consequence, inhibition of PTP1B *in vivo* is a promising strategy for the treatment of diabetes, obesity and cancer. T cell PTP (TCPTP) has been shown to be associated with some inflammatory disorders such as type 1 diabetes, rheumatoid arthritis and Crohn’s disease [11]. However, TCPTP shares the identical catalytic site and a 74% sequence identity with PTP1B, while knockout of TCPTP has proven lethal to mice [12]. Therefore, the chemical tools that achieve high selectivity between these two PTPs are admirable for the delineation of their unique role in cell physiology as well as for probing into their therapeutic potential.

Three isoforms of cell division cycle 25 (CDC25), namely CDC25A, CDC25B and CDC25C which belong to the class III cysteine-based PTPs, are identified in the human genome. They activate cyclin-dependent kinases (CDKs) and play different roles in the cell cycle regulation of mammals. However, overexpressed CDC25A and CDC25B phospatases were identified in various human cancer cells including breast, gastric, colon, ovarian and thyroid, whereas CDC25C was found to be expressed in a lower level in prostate cancers [13]. This implies that inhibitors of CDC25 phosphatases may become promising anticancer agents. Src homology-2 domain containing protein tyrosine phosphatase-2 (SHP-2) is the first virtually indentified oncogene among the PTPs, which is a mediator of cell signaling by transductions of growth factor and cytokine pathways. The identification of SHP-2 mutations in childhood and adult leukemia, and a number of solid tumors underscores its critical pathological role [14]. Moreover, Lymphoid-specific tyrosine phosphatase (Lyp) is associated with autoimmune diseases including type I diabetes and rheumatoid arthritis and abnormalities in the receptor tyrosine phosphatase CD45 have been linked to autoimmune disease. Polymorphisms in the receptor PTPsigma (PTPRS) gene are associated with ulcerative colitis [15], while STEP, a brain-specific phosphatase, was recently reported to be involved to the adverse effects of soluble amyloid-β-protein on synaptic function, and to the pathology of Alzheimer’s disease. Down-regulation of STEP blocks the synaptotoxic effects of amyloid-β, suggesting that inhibition of STEP may be a therapeutic strategy for Alzheimer’s disease [16, 17]. The widely expressed DEP1 enzyme has also received attention as a novel drug target for anti-thrombotic [18] and anti-angiogenesis therapy [19].

The compelling biochemical evidence above-depicted has resulted in a plethora of efforts by academia and the pharmaceutical community toward the production of PTPs inhibitors [20, 21]. However, among the numerous inhibitors indentified during the past decade, only a few candidates have entered clinical trials while no commercial drugs have been approved till date due mainly to their insufficient selectivity over homologous PTPs and especially, limited bioavailability. As a consequence, further efficient discovery of chemical entities competent for PTPs inhibition with enhanced selectivity and bioavailability remains desirable.

Taking nature’s cue for the creation of her primary metabolites such as polypeptides, polynucleotides and polysaccharides, Sharpless and co-workers coined the notion of ‘Click Chemistry’ early this century [22]. Unlike the conventional synthetic approaches which mainly rely on the relatively harsh carbon-carbon ligation reactions concomitant with tedious laboratorial workup, click chemistry advocates the modular and more efficient construction of useful compound libraries by taking advantage of the simpler and more selective carbon-heteroatom ligation syntheses. During the past decade, Cu(I)-catalyzed azide-alkyne 1,3-dipolar cycloaddition reaction (CuAAC) [23, 24, 25] in forming uniquely the 1,4-disubstituted 1,2,3-triazolyl derivatives, which represents the best paradigm of click chemistry, has fueled substantially refreshed energy into various fields of the modern science. Most importantly, after optimizations in reaction conditions and catalyst choices [26, 27], this chemical tool has fulfilled the efficient discovery of numerous bioactive triazolyl small-molecules for a multitude of drug targets [28-32].

More recently, CuAAC has been introduced into the modular development of PTPs inhibitors, leading to the fruitful validation of new promising leading compounds that may serve as chemical tools for the study of PTPs-related chemical biology and pharmacology. We therefore highlight herein comprehensively the advancement with respect to this emerging and exciting area.

## 3 CLICK-DERIVED PTPS INHIBITORS

The rationale behind the development of PTPs inhibitors fundamentally focuses on the identification of nonhydrolyzable phosphotyrosine (pTyr) mimetics that may bind competitively to the PTP catalytic site [20, 21, 33]. However, sole pTyr surrogates fail to achieve high binding affinity with their target PTPs and are essentially short of selectivity over the various homologous PTPs sharing the highly conserved active site. Fortunately, subsequent biochemical studies revealed that the residues flanking the pTyr are also crucial for the substrate-specific recognition of PTPs, thereby leading to a new criterion of developing PTPs inhibitors, that is, bidentate ligands that engage both the catalytic site and a peripheral less-conserved subpocket [34]. This criterion has then demonstrated particularly suited for the development of potent and selective PTPs inhibitors. Therefore, the advent of a synthetic tool that may powerfully connect pTyr mimetics with a diverse range of other functional groups having distinct structures and properties becomes necessary.

**Fig. (1):**
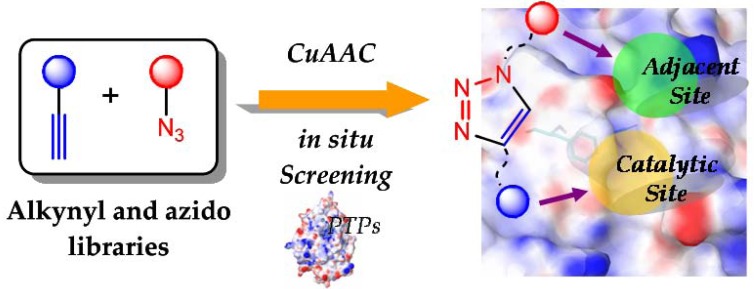
The CuAAC approach for the modular construction of bidentate PTPs inhibitors.

CuAAC, the representative of click chemistry, is deemed to act as the most efficacious chemical ligation tool for building bioactive compound libraries because of its superior stereoselectivity and functional group tolerance, and capacity to give pure products for* in situ* drug screening. These compelling merits which thoroughly complement the above-mentioned criterion prompted researchers to synthesize bidentate PTPs inhibitors via CuAAC (Fig. **1**).

**Fig. (2):**
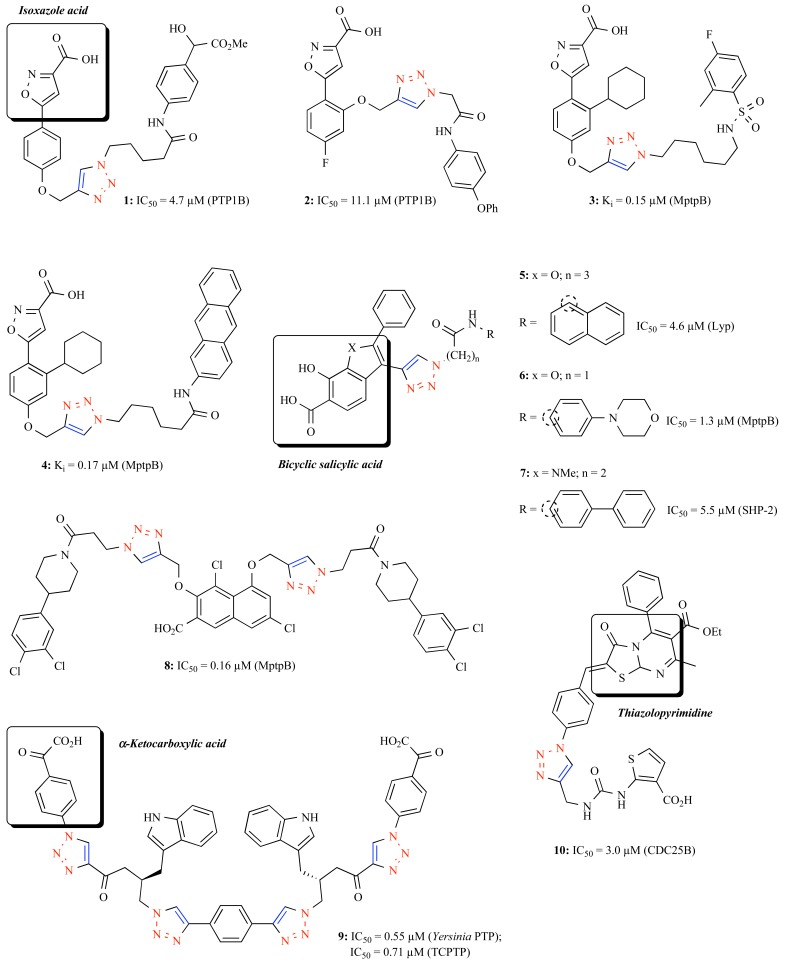
Representative triazolyl PTPs inhibitors produced by high-throughput amenable CuAAC.

In 2006, Yao and co-workers reported the first high-throughput amenable CuAAC assembly of a 66-member library comprising triazole-linked bidentate compounds for *in situ* screening on a panel of PTPs [35]. Using the isoxazole acid that has favorable cellular activity and pharmacokinetics identified by Abbott’s laboratory [36] as the pTyr mimetic, compound **1** (Fig. **2**) with an additionally ‘clicked’ tyrosine-like tail was characterized as a new PTP1B inhibitor with a comparable IC_50_ value (4.7 μM) to that of the original Abbott’s inhibitor and 5 to 160-fold selectivity over other homologous PTPs tested [37]. The same researchers then described a solid-phase strategy for the productive synthesis of azide libraries suitable for CuAAC assembly [38]. Subsequent *in situ* screening on PTP1B identified compound **2** (Fig. **2**) featuring an isoxazole acid warhead in connection with a triazolyl *O*-phenyl benzamide tail as a micromolar PTP1B inhibitor from the 325-member combinatorial library within only a week.

This approach then unprecedentedly allowed the modular CuAAC assembly of an enormous library of –3500 bidentate triazolyl isoxazole acid derivatives. Subsequent* in situ *screening led to the identification of some nanomolar MptpB inhibitors such as compounds **3** (IC_50_ = 0.15 μM) and **4** (IC_50_ = 0.17 μM) bearing a fluorobenzosulfonamide and an anthracene tail, respectively [39]. In addition, these compounds which are among the most potent MptpB inhibitors identified thus far, have remarkable selectivity over other PTPs tested (11 to 52-fold). A modeling study suggested that their isoxazole acid precursor might occupy the catalytic site of MptpB, with the triazole-connected secondary groups projecting into a peripheral site, thereby indicating the bidentate nature of the inhibitors.

As the majority of the phosphate-based pTyr mimetics encounter drawbacks such as high polarity which may lower their cellular activity, Zhang and co-workers identified, for the first time, bicyclic salicylic acid derivatives that carry sufficient polar and nonpolar interactions with the active site and yet possess improved pharmacological properties [40]. Using this drug-like precursor as the pTyr surrogate, a range of CuAAC-oriented combinatorial libraries were efficiently constructed. Compound **5** with a triazole-linked naphthalene tail was first identified from the screening of an 80-member focused click library as a new Lyp inhibitor with micromolar potency (4.6 μM) and moderate-to-good selectivity over a range of homologous PTPs examined [41]. Crystal structure of Lyp in the presence of **5** reveals that the enzyme-inhibitor interaction occurs in both the catalytic site and a cluster of unique residues adjacent to the active site. Furthermore, this inhibitor with excellent cellular activity also helped delineate the previously unexploited conformational change of Lyp. Since biochemically selective MptpB inhibitors with *in vivo* activity are elusive, the researchers further identified compound **6** flanking a triazolyl 4-phenylmorpholine tail from the 80-member click library as a unique MptpB inhibitor [42]. This compound merits its good selectivity (at least 11-fold over other PTPs) and highly efficacious cellular activity, which has been subsequently used as a chemical tool for demonstrating that inhibition of MptpB in macrophages prevents tuberculosis (TB) growth in host cells. These new data wonderfully support the notion that specific inhibitors of MptpB may serve as effective antiTB therapeutics.

Considering that the indole nucleus are a class of prominent scaffold found in numerous natural products with vital medicinal value [43], Zhang and co-workers developed a novel indole-based bicyclic salicylic acid derivative for creation of a 212-member CuAAC library [44]. Five hits with micromolar potency were identified as specific SHP-2 inhibitors, in which **7** (IC_50_ = 5.5 μM) exhibits the best potency and is highly active on cellular level. Interestingly, despite the indole-based salicylic precursor occupies the catalytic site, kinetic and crystal studies established that **7** is a noncompetitive inhibitor and prevents the closure of WPD loop that is conventionally required for the catalytic activity of the PTPs. X-ray structure of **7**-SHP-2 further implies that the distal biphenyl group of **7** makes additional hydrophobic contacts with a region of SHP-2 highly divergent among the PTPs, thereby enhancing its potency and specificity. A unique ‘double click’ strategy was recently proposed by Zhang, which aims at coupling two functional groups with a core pTyr mimetic for further increasing interactions with the subpockets of PTPs [45]. Consequently, various azides were ‘clicked’ onto five different dipropargyl salicylic acid scaffolds in generating a 212-member library, from which tridentate compound **8** (IC_50_ = 160 nM) was disclosed as a new potent MptpB inhibitor. Modeling study predicted that the two additional dichlorophenyl piperidine groups of **8** may generate interactions with, respectively, two unique subpockets of MptpB, thereby reasoning its superior selectivity (>25-fold over all mammalian PTPs).

Xie and Seto reported a two-stage CuAAC library based on α-ketocarboxylic acid as the pTyr mimetic [46]. They discovered from the first round click assembly that a single α-ketoacid contained bis-triazole exhibits parallel inhibitory potency on three PTPs, namely *Yersinia* PTP, PTP1B and TCPTP. A second round click assembly then led to the identification of **9** having two α-ketoacid precursors bridged through four triazoles as a submicromolar *Yersinia* PTP (IC_50_ = 0.55 μM) and TCPTP (IC_50_ = 0.71 μM) dual inhibitor with enhanced selectivity over other PTPs tested.

The chemical entities identified serving as CDC25B inhibitors mainly fall into quinone derivatives that irreversibly bind with the protein *via *arylation of its nucleophilic cysteine center [6, 13]. However, these compounds may also release reactive oxygen species (ROS), causing toxicity to normal tissues. In an effort to tackle this issue, Duval and co-workers identified benzylidene-thiazolopyrimidines (BTP) as novel heterocyclic inhibitors of CDC25 enzymes and subsequently built an 87-member triazolyl BTP (TBTP) library *via* CuAAC [47]. The structure activity relationship (SAR) analysis indicated that *p*-TBTPs are better inhibitors over the (*o*,*m*)-TBTP counterparts, while in the series of the former, inhibitors featuring aryltriazole moieties are privileged. Compound **10** which is the sole exception with a triazole-linked heterocyclic carboxylic acid tail exhibited the best inhibitory activity (IC_50_ = 3.0 μM) among the library, probably by generating an additional hydrogen-bond network with the appropriate enzymatic surface of CDC25B.

Sugars ubiquitously exist in nature, governing myriad pivotal biological and pathological events through specific sugar-protein recognitions. Due to their well-defined stereo-structure and high biocompatibility, these abundant natural primary metabolites have received particular interest in modern drug discovery [48, 49]. The identification of tamiflu that is structurally originated from sialic acid (a monosaccharide) in efficiently combating influenza may potently exemplify the immense potential of sugar scaffolds in drug design and development [50].

**Fig. (3):**
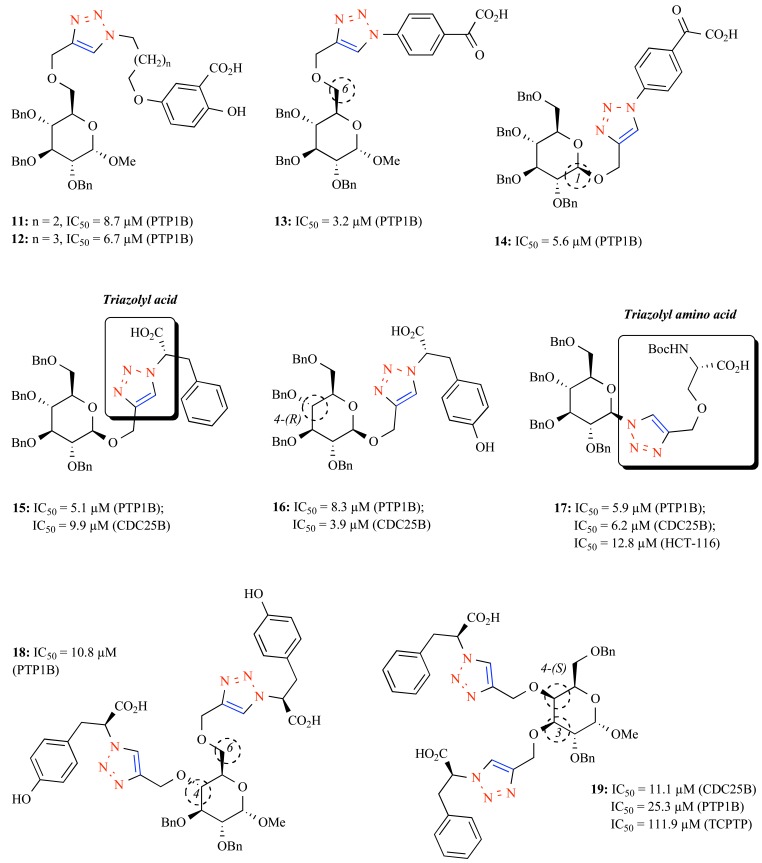
Sugar scaffold-based triazolyl PTPs inhibitors prepared by CuAAC.

Enlightened by a recent report where isolated glycosyl salicylic acid derivatives were identified as good PTP1B inhibitors [51], we prepared triazole-linked salicylic acid glycosides for evaluation of their inhibitory potency on PTP1B [52]. However, these glycoconjugates with hydroxyl-free sugar moieties exhibited weak inhibition toward PTP1B, due likely to the absence of tailed aryl groups. A novel series of sugar-salicylic acid hybrids were subsequently synthesized by CuAAC, in which compounds **11** (IC_50_ = 8.7 μM) and **12** (IC_50_ = 6.7 μM) having a benzylated glucosyl moiety were identified as low micromolar PTP1B inhibitors with certain selectivity over other PTPs tested (3-fold over TCPTP and 8 to 15-fold over SHP-1, SHP-2 and [LAR leukocyte antigen-related protein]) (Fig. **3**) [53]. The binding behavior of these compounds with PTP1B was proposed by a modeling study, revealing that the salicylic acid precursor may enter the catalytic site and the benzyl glucoside makes hydrophobic interactions with the YRD motif.

Enlightened by Seto’s study, the α-ketocarboxylic acid warhead was used as a pTyr mimetic for coupling with benzyl sugar templates *via* CuAAC [54, 55]. Compounds **13** (IC_50_ = 3.2 μM) and **14** (IC_50_ = 5.6 μM) bearing the α-ketoacid moiety on the C6- or C1-position of the sugar core were identified as new PTP1B inhibitors (selectivity of **13**: 4.5-fold over TCPTP and >31-fold over SHP-1, SHP-2 and LAR) (Fig. **3**) [54]. Compared to salicylic acids, the α-ketoacid warhead, as predicted by a modeling study, could more deeply extend to the narrow active site of PTP1B due to the existence of an additional keto group. Furthermore, the benzyl sugar moiety was suggested to make nonpolar contacts with partial second pTyr site of the enzyme that differ from other PTPs by several amino acids [56].

More recently, we proposed that triazolyl acids and triazolyl amino acids (Fig. **3**) could serve as a new range of pTyr mimetics because of their structural proximity to Abbott’s isoxazole acid (Fig. **2**). As a consequence, various readily available azido and alkynyl amino acids were coupled with alkynyl or azido sugar templates *via* CuAAC, leading to the formation of novel triazolyl peptidomimetics with rich structural diversities. By clicking four azido amino acids onto benzyl propargyl glycosyl templates, the first series of triazolyl acid derivatives were acquired [57]. Biological assay on a panel of PTPs revealed that the tyrosine and phenylalanine derivatives possess better activity than their serine and threonine counterparts, meaning that the additional aryl groups of the former are beneficial. Compounds **15** and **16** were identified as micromolar PTP1B and CDC25B inhibitors, albeit with low-to-moderate selectivity over other PTPs (1.5 to 7-fold over TCPTP, SHP-1 and SHP-2). Notably, the C4-epimer of **16** showed 3-fold decreased potency on CDC25B, indicating that the configurational change in the benzyl sugar moiety may vary the activity. For building the second series, alkynyl L-serine and L-threonine were clicked onto various azido benzyl sugar templates, affording triazolyl amino acid derivatives [58]. Compound **17** bearing a Boc-L-serine precursor was identified as a competitive PTP1B inhibitor and a mixed-type CDC25B inhibitor with 2 to 17-fold selectivity over other PTPs, which also displayed reasonable cytotoxicity on HCT-116 (human colon cancer cell line). However, debenzylation of **17** led to largely decreased activity, demonstrating the crucial role of the distal benzyl groups for obtaining desirable binding affinity with the PTPs.****

A dual click strategy was also employed to build the third series of bidentate amino acid-sugar conjugates. Azido tyrosine and phenylalanine were clicked simultaneously onto the two different adjacent positions of a dipropargyl monosaccaride template, presumably assessing both the first and second sites of PTP1B [59]. Biological assay revealed that the activity of these compounds is largely dependent on the substitution position of the triazolyl acid residue on the sugar scaffold. The C3,4- and C2,6-modified conjugates were impotent whereas their C4,6-and C2,3-modified counterparts showed moderate inhibitory potencies (IC_50_ = 10 to 20 μM) on PTP1B. The C4,6-modified glucosyl tyrosine derivative **18** (IC_50_ = 10.8 μM) is the most potent inhibitor among this series, which, however, is less active than the mono-triazolyl amino acid-sugar conjugates. Subsequently performed modeling study suggested that the two triazolyl acid residues of **18** do have the propensity to approach both the first and the second site of PTP1B, but their mutual interactions are weak due to the inadequate molecular length. This offers a rationale for future structural optimizations. Since the naturally defined epimeric identity of sugars endows them with distinct recognition response toward proteins [60], we envisaged to introduce the triazolyl acids directly onto the epimeric sites of glucose and galactose, a pair of C4-epimers, for estimating whether the resulting inhibitory activity would be different [61]. Interestingly, whereas the galactosyl bis-triazolophenylalanine derivative **19** with a C4-axial bond showed micromolar inhibitory potency (IC_50_ = 11.1 μM) on CDC25B, its C4-epimer with an equatorial bond was determined inactive on the same target.

## 4 SUMMARY AND PERSPECTIVE

Since the definition of click chemistry by Sharpless and co-workers, considerably much energy has been infused to the progress of medicinal chemistry as witnessed by the past decade of the third millennium. By taking advantage of the extraordinarily modular CuAAC, combinatorial libraries that contain tens to thousands of parallel compounds were able to be created efficaciously in a high throughput manner for *in situ* drug screening.

Meanwhile, despite only for six years has CuAAC been introduced into the development of PTPs inhibitors, the achievements made by the researchers are encouraging. From Yao’s first focused library to his large-scale 3500-member collection, diverse triazolyl PTPs inhibitors with admirable potency and specificity have been unprecedentedly disclosed. Zhang’s elegant crystallographic contribution further substantiated the fact that by clicking different aromatic tails making hydrophobic contacts to the divergent PTP subpockets with even a same pTyr mimetic core, selectivity among the homologous PTPs could be easily achieved. Additionally, most of these triazolyl inhibitors developed have proven efficacious either as chemical tools for the study of the PTPome or as drug candidates that merit promising cellular activity and pharmacological properties. We also proposed triazolyl glycopeptidomimetics which are essentially constituted by the highly biocompatible amino acids and sugars as a new range of chemical scaffolds serving as PTPs inhibitors, providing alternative insights into the field.

The essential flaws that hamper the validation of the PTPs-targeted drug candidates for the treatment of various human diseases are the insufficient selectivity and bioavailability of the currently developed inhibitors. Apparently, the CuAAC click chemistry, a concise but powerful chemical ligation tool, has substantially boosted the acquisition of new competent PTPs inhibitors with, simultaneously, desired selectivity as well as promising pharmacological profiles. Prompted by this, we are confident to continuously witness the immense contribution of this ingenious synthetic tool toward the acceleration of the PTP-targeted drug discovery.
